# Regulierung des Schattenbankensektors: Der Fall des chinesischen BigTech Ant Group als Blaupause?

**DOI:** 10.1007/s41025-021-00229-0

**Published:** 2021-06-24

**Authors:** Victoria Böhnke

**Affiliations:** Institut für Kreditwesen, Westfälische Wilhelms-Universität Münster, Münster, Deutschland

**Keywords:** Bank, China, Finanzregulierung, Finanzstabilität, Schattenbank, Bank, China, Financial regulation, Financial stability, Shadow bank, G21, G28

## Abstract

Der kurzfristig abgesagte Börsengang der Ant Group im November 2020 hat für Aufsehen an den internationalen Finanzmärkten gesorgt. Ant Group ist das auf Finanzdienstleistungen spezialisierte Tochterunternehmen eines großen chinesischen Technologiekonzerns. Eine Regulierungsänderung, die die Ant Group als Finanzholding einstuft und das systemische Risiko seiner Geschäftsaktivitäten begrenzen soll, zeigt, wie die chinesischen Behörden die Marktmacht großer Technologiekonzerne im Finanzsektor einschränken und so eine stärkere Regulierung des Schattenbankensektors umsetzen. Kann dieser Ansatz zur Regulierung des Schattenbankensektors in China ein Vorbild für die Europäische Union sein? Zunächst werden die Entwicklung des Schattenbankensektors und seine Bedeutung für globale Finanzstabilität sowie aktuelle Herausforderungen für die Regulierung diskutiert. Anhand einer Fallstudie zur Ant Group werden Chancen und Herausforderungen des Regulierungsansatzes der chinesischen Behörden erörtert und Implikationen für die Regulierung in Europa abgeleitet. Zusammenfassend erscheint eine Regulierung großer Technologiekonzerne, die umfangreiche, mit traditionellen Banken vergleichbare Finanzdienstleistungen anbieten, mit Blick auf die Finanzstabilität erforderlich, da diese über eine (potenziell) große Marktmacht verfügen.

## Abstract (en)

Cancelling the Ant Group Initial Public Offering on short notice in November 2020 attracted attention on the international financial markets. Ant Group is the subsidiary of a large Chinese technology corporation that specialises in financial services. A regulation change that classifies Ant Group as a financial holding company and which aims to curb the systemic risk of its business activities shows how the Chinese authorities limit the large technology corporations’ market power and thereby introduce a stricter regulation of the shadow banking sector. Can this approach to regulate the Chinese shadow banking sector serve as a model for the European Union? This paper first discusses the development of the shadow banking sector, its relevance for global financial stability, and current challenges for its regulation. Then, based on a case study of Ant Group, this paper discusses opportunities and challenges of the Chinese authorities’ regulatory approach and derives implications for the regulation in Europe. In summary, a regulation of large technology corporations that offer a large range of financial services comparable to traditional banks seems necessary with reference to financial stability, as these companies (potentially) have extensive market power.

## Einführung

Innovation im Bankensektor wird insbesondere durch junge Unternehmen angetrieben, die technologiebasierte, besonders kundenorientierte Finanzdienstleistungen anbieten (Philippon 2019, S. 13–14). Ebenso haben in den letzten Jahren einige einflussreiche Technologieunternehmen mit einem sehr großen bereits existierenden Kundenstamm, auch *BigTech *genannt, ihre Geschäftstätigkeit in den Bereich der Finanzdienstleistungen ausgeweitet (Frost et al. 2019, S. 762). In der Folge wird Finanzintermediation nicht mehr vor allem von traditionellen Banken durchgeführt, sondern auch sogenannte Schattenbanken^1^ übernehmen originäre Bankfunktionen (Buchak et al. 2018, S. 2). Während diese Schattenbanken aus der Perspektive einer traditionellen Bank einen gefährlichen Wettbewerber darstellen, stellen sie die Regulierung des Finanzsektors vor ebenso große Herausforderungen (Adrian und Jones 2018, S. 14–16; Philippon 2019, S. 9–13). Denn wenn eine Regulierung des Sektors aus bestimmten Gründen^2^ von Bedeutung ist, sollte diese für alle Unternehmen gelten, die Finanzdienstleistungen anbieten, um Wettbewerbsverzerrungen zu vermeiden (Zetzsche et al. 2017, S. 35).

Gerade mit der stetigen Entwicklung neuer Innovationen entwickelte sich ein Angebot von Finanzintermediation außerhalb des traditionellen Bankensystems, welches nicht der regulären Bankenregulierung unterliegt. Stattdessen müssen finanzielle Innovationen zuerst als solche erkannt und falls nötig mit speziellen Regulierungsanforderungen adressiert werden. Das Financial Stability Board (FSB) hat beispielsweise Peer-to-Peer-Lending^3^ als eine der bedeutendsten Innovationen der letzten Jahre identifiziert und berichtet, dass einige Länder in diesem Bereich inzwischen Regulierungsanforderungen verabschiedet haben (Financial Stability Board 2020c, S. 6). Aus der Sicht der globalen Finanzstabilität gilt insbesondere dem wachsenden Einfluss der BigTech mit zunehmendem Angebot von Finanzdienstleistungen besondere Aufmerksamkeit. Erste Erfahrungen beispielsweise aus China, wo sich bereits große Technologiekonzerne in diesem Bereich etabliert haben, zeigen, wie eine Entwicklung in Zukunft auch in anderen Volkswirtschaften aussehen könnte und werfen dabei Fragen nach ihren Auswirkungen auf die Finanzstabilität auf (Frost et al. 2019, S. 781–782; Financial Stability Board 2020a, S. 4–7).

In diesem Zusammenhang sorgte zuletzt der knapp 48 h vor dem geplanten Termin abgesagte Börsengang des chinesischen Unternehmens *Ant Group* für Aufsehen (The Economist 2020a). Die Ant Group gehört zur chinesischen Alibaba Group, einem der größten Technologiekonzerne der Welt. Die seit 2014 an der New Yorker Börse gelistete Alibaba Group ist Eigentümerin von drei großen chinesischen E‑Commerce-Plattformen für die Bereiche Business-to-Business (B2B), Business-to-Consumer (B2C) sowie Consumer-to-Consumer (C2C) und erzielte 2020 einen Börsenwert von mehr als 600 Mrd. € (Gambacorta et al. 2020, S. 27–28). Der Technologiekonzern gründete die Ant Group 2014 als Mutterunternehmen für seinen Zahlungsdienst *Alipay*, ursprünglich unter dem Namen „Ant Financial Services Group“ (Alibaba Group 2020, S. 8–11; Gambacorta et al. 2020, S. 27–28). Alipay bietet, ähnlich wie Paypal, eine Online-Zahlungsplattform an. Zusätzlich hat das Unternehmen eine auf QR-Codes basierende Technologie für Offline-Anbieter entwickelt. Mit dieser Technologie stellt Alipay seit Mitte 2017 einen mobilen Bezahldienst bereit, der Zahlungen per Smartphone ermöglicht und das Bargeld in China fast vollständig abgelöst hat (Dathe und Helmold 2020; Gambacorta et al. 2020, S. 27–28). Darüber hinaus bietet die Ant Group inzwischen auch technologiebasierte Finanzdienstleistungen im Bereich Kreditvergabe und Kapitalanlagen an und ist damit eines der am weitesten entwickelten BigTech im Finanzbereich weltweit (Dathe und Helmold 2020; Gambacorta et al. 2020, S. 2).

Der für den 5. November 2020 geplante Börsengang der Ant Group in Hongkong und Shanghai wurde am 3. November aufgrund von kürzlich vorgenommenen Regulierungsänderungen durch die chinesische Regulierungsbehörde ausgesetzt (The Economist 2020a; Trivedi 2020). Mit den neuen Regelungen schreiben die chinesischen Regulierungsbehörden unter anderem vor, dass mindestens dreißig Prozent eines Kredits durch den Kreditgeber selbst refinanziert werden müssen. Im Fall der Online-Kreditvergabe durch Tochterunternehmen der Ant Group, die in ihrer überwiegend in Kooperation mit Banken durchgeführten Kreditvergabe deutlich weniger als dreißig Prozent auf der eigenen Bilanz führen, entspricht ein Großteil der Transaktionen somit nicht den neuen Vorgaben. Zusätzlich könnten neue Offenlegungsvorschriften die zukünftige Zusammenarbeit der Ant Group mit Banken erschweren (Dathe und Helmold 2020; The Economist 2020a).

Der Fall zeigt, wie die chinesischen Regulierungsbehörden die Finanzaktivitäten großer Technologiekonzerne in den Blick nehmen, indem sie ihre auf Finanzdienstleistungen spezialisierten Tochterunternehmen anhand eines Kriterienkatalogs als Finanzholding einstufen. Durch gezielte regulatorische Vorgaben wird sichergestellt, dass Unternehmen selbst einen Teil des Risikos tragen müssen („Skin in the game“) und somit ein ausreichendes Eigeninteresse daran haben, ihr Risiko zu begrenzen (Chemla und Hennessy 2014, S. 1597; Wah et al. 2020; The Economist 2021). Dieser Schritt kann, sofern die Regulierungsvorschriften auch in Zukunft effektiv umgesetzt werden, einen entscheidenden Schritt zur erfolgreichen Regulierung des chinesischen Schattenbankensektors darstellen (Wah et al. 2020). Die chinesischen Regulierungsbehörden setzen damit Empfehlungen des Internationalen Währungsfonds (IWF) aus der zuletzt 2017 durchgeführten Überprüfung des chinesischen Finanzsektors um (Internationaler Währungsfonds 2017, S. 29–32, 2019c, S. 80–95).

In der Europäischen Union (EU) wird der Einfluss des Schattenbankensektors zwar überwacht und eine Reihe einzelner Maßnahmen wurden bereits verabschiedet, ein umfassendes makroprudenzielles Maßnahmenpaket fehlt jedoch bislang (Europäische Kommission 2012; Europäischer Ausschuss für Systemrisiken 2020, S. 99–100; Hodula et al. 2020, S. 2). Trotz der sehr großen Unterschiede zwischen den Finanzsystemen in China und Europa ist es durchaus denkbar, dass große Technologiekonzerne, ähnlich wie in China, auch in Europa noch stärker im Finanzsektor aktiv werden und dadurch ein wachsendes Risiko für die Stabilität des Finanzsystems darstellen. Vor diesem Hintergrund stellt sich die Frage nach einer Generalisierbarkeit des Regulierungsansatzes der chinesischen Behörden. Kann die Regulierung des Schattenbankensektors in China ein Vorbild für die EU sein? Im Folgenden werden zunächst die Entwicklung des globalen Schattenbankensektors und seine Bedeutung für Finanzstabilität sowie aktuelle Herausforderungen in der Regulierung des Schattenbankensektors diskutiert. Vor dem Hintergrund einer Fallstudie zur Ant Group werden schließlich Implikationen für die EU analysiert.

## Die Entwicklung des Schattenbankensektors und seine Bedeutung für globale Finanzstabilität

Auf globaler Ebene rückte der Schattenbankensektor nach der Finanzkrise 2008 als einer der Auslöser der Krise in den Fokus des öffentlichen Interesses.^4^ Aufgrund des enormen Einflusses des Sektors auf die globale Finanzstabilität erarbeitete das FSB gemeinsam mit dem Baseler Ausschuss für Bankenaufsicht und der Internationalen Organisation der Wertpapieraufsichtsbehörden erste Regulierungsvorschläge als Grundlage für die nationalen Regulierungsbehörden (Financial Stability Board 2013b, S. 3–5; Michler 2016, S. 146). In den seit 2011 regelmäßig erscheinenden Monitoring Reports überwacht das FSB die Entwicklung des globalen Schattenbankensektors, präsentiert aktuelle Trends und dokumentiert potenzielle Risiken für globale Finanzstabilität (Financial Stability Board 2020b, S. 3–5).

**Figure 1:**
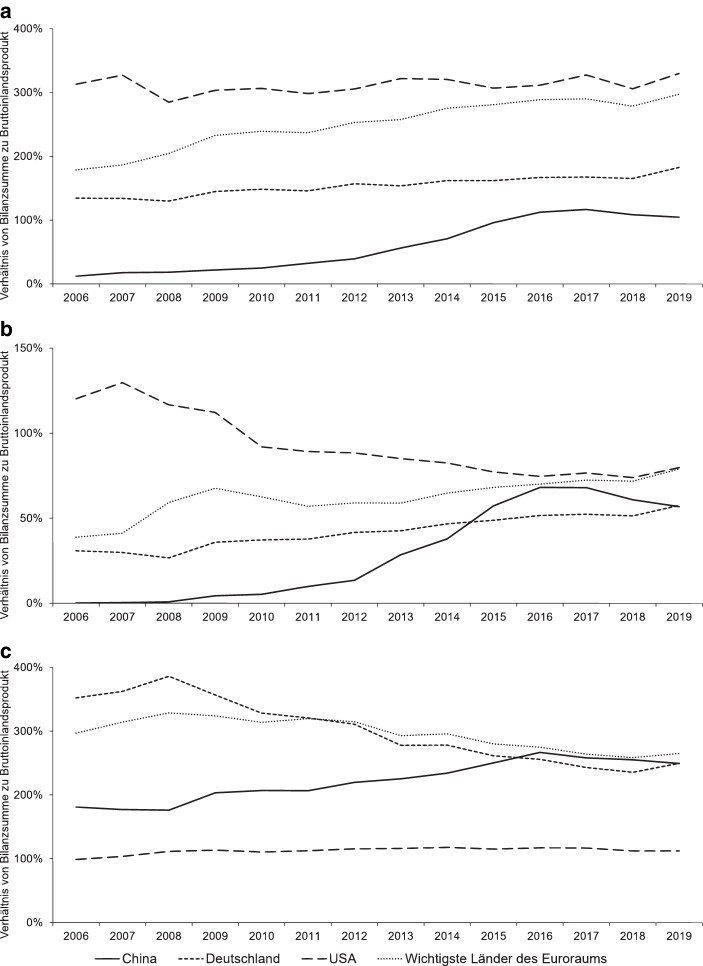

Teil (a) von Abb. 1 veranschaulicht die Entwicklung der nationalen Schattenbankensektoren^5^ im Verhältnis zum Bruttoinlandsprodukt (BIP) für China, Deutschland, die USA sowie für die wichtigsten Länder des Euroraums: Belgien, Deutschland, Frankreich, Irland, Italien, die Niederlande und Spanien. Seit der Finanzkrise ist das Verhältnis der aggregierten Bilanzsumme des Schattenbankensektors zum nationalen BIP lediglich in den USA, dem Land mit dem größten Gesamtvolumen des Sektors in US-Dollar, und in Deutschland annähernd auf dem gleichen Niveau geblieben beziehungsweise leicht angestiegen. In den Ländern des Euroraums – maßgeblich beeinflusst durch den starken Anstieg in Irland – und in China hingegen ist das Verhältnis von Bilanzsumme zu BIP vor allem im Zeitraum von 2009 bis 2016 deutlich gestiegen (Financial Stability Board 2021).

Zusätzlich zu diesem umfassenden Maß zur Erfassung aller Schattenbankenaktivitäten, betrachtet das FSB speziell den Schattenbankensektor im engen Sinne („narrow measure“) als „Nicht-Bank-Finanzintermediation, die potenziell ein systemisches Risiko für das Finanzsystem darstellt“ (Financial Stability Board 2014, S. 6).^6^ Teil (b) von Abb. 1 zeigt die Entwicklung des Schattenbankensektors im engen Sinne im Verhältnis zum nationalen BIP ebenfalls für China, Deutschland, die USA und die wichtigsten Länder des Euroraums. Das Verhältnis der Bilanzsumme des Schattenbankensektors im engen Sinne zum BIP verdeutlicht seine große Bedeutung in den USA im Zeitraum der Finanzkrise und die anschließende Abnahme im Zeitverlauf. In Europa steigt das Verhältnis der Bilanzsumme des Schattenbankensektors im engen Sinne zum BIP zwar leicht an, insbesondere in Deutschland bleibt die Bilanzsumme jedoch unter sechzig Prozent des BIP.

In China dagegen ist ein rasantes Wachstum des Schattenbankensektors mit besonderer Relevanz für die Finanzstabilität für den Zeitraum von 2012 bis 2016 erkennbar. In absoluten Zahlen versiebenfachte sich das Volumen in China innerhalb von nur vier Jahren und stabilisierte sich seit 2017 mit dem zweithöchsten Volumen in US-Dollar nach den USA (Financial Stability Board 2021). Im Vergleich zu den Schattenbankensektoren entwickelter Volkswirtschaften wie den USA, umfasst der Schattenbankensektor in China nicht nur Finanzintermediation durch Nicht-Banken, sondern auch einen eng mit den Geschäftsbanken verbundenen Schatten der Banken, engl. „Banks’ shadow“ (Sun 2019, S. 4). Zu diesem Teil des chinesischen Schattenbankensektors gehören Aktivitäten der Geschäftsbanken, die der Realwirtschaft finanzielle Mittel zur Verfügung stellen, ohne diese als Kredit in ihrer Bilanz aufzuführen. Banken umgehen dabei regulatorische Vorgaben, indem sie beispielsweise bilanzielle Maßnahmen ergreifen, die nicht dem Standard entsprechen. Diese Aktivitäten stellen ein besonderes Risiko für die Finanzstabilität dar, weil sie die Effektivität der Geldpolitik und der Regulierung beeinträchtigen (Sun 2019, S. 1–6; Acharya et al. 2021, S. 6–10).

Der Entwicklung der Schattenbankensektoren stellt Teil (c) von Abb. 1 jeweils das Verhältnis der Bilanzsumme des traditionellen Bankensektors zum BIP gegenüber. Während die Bilanzsumme des Bankensektors im Verhältnis zum BIP in den USA auf einem Niveau bleibt, sinkt das Verhältnis in den wichtigsten Ländern des Euroraums seit 2008 deutlich. In China hingegen nimmt die aggregierte Bilanzsumme in Prozent des BIP zu und zeigt, ähnlich wie die Entwicklung des Schattenbankensektors, eine wesentliche Steigerung der finanziellen Aktiva im Verhältnis zum nationalen BIP seit 2009 sowie eine Stabilisierung seit 2017 (Financial Stability Board 2021).

Die Marktturbulenzen im März 2020, anders als 2008 ausgelöst durch den externen Schock der Covid-19-Pandemie, haben die Widerstandsfähigkeit des globalen Finanzsystems erneut auf die Probe gestellt. In seiner ganzheitlichen Beurteilung kommt das FSB zu dem Ergebnis, dass vor allem im Bereich der Nicht-Bank-Finanzintermediation weitere Regulierungsanstrengungen notwendig sind, um Finanzstabilität sicherzustellen (Financial Stability Board 2020d, S. 42–43). Auch der IWF beurteilte den stetig wachsenden globalen Schattenbankensektor bereits vor Beginn der Covid-19-Verwerfungen als zentralen Risikofaktor für globale Finanzstabilität (Internationaler Währungsfonds 2019a, S. 6–7, 2019b, S. 4–6). Die jüngsten Marktturbulenzen offenbarten insbesondere Schwachstellen durch grenzübergreifende Verflechtungen zwischen Banken und Schattenbanken, die zu einer Verstärkung der negativen Auswirkungen der Pandemie auf den internationalen Finanzmärkten führten (Aldasoro et al. 2020, S. 68). Für die Reformagenda nach dem Ende der Pandemie identifiziert der IWF den Schattenbankensektor neben den Risiken aus dem anhaltenden Niedrigzinsumfeld als einen der beiden zu adressierenden Bereiche (Internationaler Währungsfonds 2020, S. 26–27).

## BigTech als Herausforderung in der Regulierung des Schattenbankensektors

Konkret nennt das FSB digitale Schattenbanken und die Kreditvergabe durch technologiebasierte Unternehmen als aktuelle Innovationen im Bereich der Nicht-Bank-Finanzintermediation (Financial Stability Board 2020c, S. 6). Unternehmen mit besonderer Bedeutung für die Finanzstabilität in diesem Zusammenhang sind BigTech, da sie bereits über eine technologiebasierte Plattform und einen sehr großen Kundenbestand, inklusive umfangreicher Kundeninformationen, verfügen und so potenziell schnell eine große Marktmacht aufbauen können (Zetzsche et al. 2017, S. 28–29; Frost et al. 2019, S. 761–765). In China, wo Aktivitäten im Schattenbankensektors gerade in den letzten Jahren deutlich zugenommen haben, sind große Technologiekonzerne dieser Art bereits etabliert. In Europa sind zwar BigTech aus den USA mit Bezahldiensten vertreten, die Marktentwicklung ist jedoch deutlich weniger fortgeschritten als in China (Frost et al. 2019, S. 761–762; Financial Stability Board 2020a, S. 4–7).

In Europa oder in den USA, wo die Nutzung von Bankkonten oder Kreditkarten weit verbreitet ist, nutzen BigTech für ihre innovativen Bezahlsysteme in der Regel die bereits existierende Infrastruktur der Banken. In Märkten ohne diese Infrastruktur, beispielsweise in China, haben BigTech dagegen eine eigene Infrastruktur für ihre Bezahlsysteme aufgebaut (Frost et al. 2019, S. 767). Begünstigt durch das fehlende Angebot traditioneller Banken, bieten chinesische BigTech inzwischen ein umfangreiches Angebot von Finanzdienstleistungen für Privatkunden und Kleinunternehmer an (Xie et al. 2016, S. 3; Hau et al. 2019, S. 62). Während diese Entwicklung maßgeblich zu einer Vertiefung und Integration des chinesischen Finanzmarktes beiträgt und der großen Landbevölkerung den Zugang zu Krediten ermöglicht, wirft sie gleichzeitig Fragen nach dem Verbraucherschutz, der Marktdominanz der BigTech und der Finanzstabilität auf (Buchak et al. 2018, S. 2; Financial Stability Board 2020a, S. 16–23).

Auf Basis der ursprünglich vom FSB vorgeschlagenen Regulierungsempfehlungen mit dem Ziel, systemische Risiken des Schattenbankensektors zu reduzieren, müssen diese Art potenzieller Risikoquellen für die Finanzstabilität zunächst ermittelt und analysiert werden (Financial Stability Board 2013a, S. 6–7). Das systemische Risiko durch die Finanzdienstleistungen der BigTech besteht, ähnlich wie im Fall anderer Schattenbankenaktivitäten, unter anderem aus fehlender Regulierung und Aufsicht. Sofern diese Unternehmen bankähnliche Finanzdienstleistungen anbieten, erscheint eine Begrenzung ihrer Risikobereitschaft, beispielsweise durch adäquate Kapitalunterlegung, notwendig, um potenzielle Verluste abzufedern.

Vor allem im Fall eines großen Technologiekonzerns, der bereits über eine umfangreiche Marktmacht verfügt, schaffen seine Größe und Vernetzung bereits beim Eintritt in den Finanzmarkt ein systemisches Risiko (Zetzsche et al. 2017, S. 28–29; Frost et al. 2019, S. 761). Hohe unerwartete Verluste können dabei als Signal für die Schwäche des gesamten Sektors interpretiert werden und weitere finanzielle Ansteckungseffekte auslösen (Financial Stability Board 2017, S. 17–25). Zusätzlich sind technologiebasierte Finanzdienstleistungen potenziell anfällig für operationelle Risiken. Insbesondere im Bereich Datensicherheit und der Verlässlichkeit neuer Technologien können Risiken auftreten, die negative Auswirkungen auf die Finanzstabilität haben (Financial Stability Board 2020a, S. 23).

Für die Regulierung stellen finanzielle Innovationen gerade durch ihre dynamische Entwicklung eine besondere Herausforderung dar (Michler 2016, S. 165–166; Ehrentraud et al. 2020, S. 4–5; Yadav 2020, S. 1109). Zwar soll die Innovationsfähigkeit eines Finanzsystems durch eine strenge Regulierung und daraus resultierenden Compliance-Kosten nicht zu stark eingeschränkt werden, eine Regulierung mit den gleichen Vorschriften für alle Anbieter von Finanzdienstleistungen („Level Playing Field“) ist jedoch wichtig, um faire Wettbewerbsbedingungen zu schaffen und Marktverzerrungen zu vermeiden (Morrison und White 2009, S. 1099; Zetzsche et al. 2017, S. 34–36; Carstens 2018, S. 4).

Bemerkenswert ist bei der Entwicklung der BigTech nicht nur die Geschwindigkeit, mit der diese Konzerne den Wandel der Finanzbranche durch Big-Data-Analysen, maschinelles Lernen und künstliche Intelligenz vorantreiben, sondern auch die stetig wachsende Anzahl und Vielfalt der Innovationen, insbesondere im Fall großer Technologiekonzerne mit bereits existierenden E‑Commerce-Plattformen (Zetzsche et al. 2017, S. 34–36). Das schnelle Wachstum dieses Schattenbankenmarktes ist dabei vor allem in den technologischen Vorteilen der BigTech und den regulatorischen Unterschieden im Vergleich zu Banken begründet (Buchak et al. 2018, S. 39).

Brummer und Yadav (2019) stellen ein Rahmenwerk für Regulierungsansätze vor, welches den Zielkonflikt der Regulierungsbehörden im Spannungsfeld zwischen finanzieller Innovation, Marktintegrität und Einfachheit der Regeln einordnet. Die Autoren argumentieren, dass in der Regulierung bestenfalls zwei der drei Aspekte erreicht werden könnten. Wenn beispielsweise Innovation gewünscht sei und Marktintegrität gefördert werden soll, bedürfe es komplexer Regeln und Ausnahmen, die durch steigende Compliance-Kosten jedoch vor allem kleinere Marktteilnehmer träfen (Brummer und Yadav 2019, S. 248–249). Da die Dynamik im Finanzmarkt unterschiedlicher Länder stark durch lokale Rahmenbedingungen geprägt ist, spielen zusätzlich nationale Unterschiede eine entscheidende Rolle (Yadav 2020, S. 1142–1143).

Potenzielle Ansätze in der Regulierung von innovativen und technologiebasierten Finanzunternehmen umfassen formelle Maßnahmen wie eine Lizenz für bestimmte Finanzdienstleistungen, aber auch informelle Maßnahmen, beispielsweise Handlungsempfehlungen oder eine sogenannte *regulatory Sandbox* (Brummer und Yadav 2019, S. 282–297). Der Begriff regulatory Sandbox beschreibt das Angebot einer Regulierungsbehörde, innovative Geschäftsideen in einer kontrollierten Umgebung zu testen. Unternehmen erhalten dabei Unterstützung bei der Einhaltung der regulatorischen Anforderungen und die Regulierungsbehörden können Risiken neuer Technologien frühzeitig erkennen und adressieren (Cornelli et al. 2020, S. 2).

Die Regulierungsansätze ermöglichen ein unterschiedliches Ausmaß an Kontrolle und Orientierungshilfe. Eine regulatory Sandbox oder informelle Handlungsempfehlungen sind dabei insbesondere bei innovativen Technologien zielführend, eine formelle Lizenzierung oder rechtliche Vorschriften dagegen eignen sich zur Regulierung ausgereifter Technologien, bei denen Risiken bereits klarer umrissen werden können (Brummer und Yadav 2019, S. 282–297). Neben Vorteilen neuer Technologien im Finanzsektor müssen die Regulierungsbehörden vor allem den Einfluss auf das Finanzsystem als Ganzes, den Verbraucherschutz und operationelle Risiken beurteilen, um adäquate Maßnahmen abzuleiten (Financial Stability Board 2020a, S. 16–23).

## Der Fall des chinesischen BigTech Ant Group

Der kurzfristig abgesagte Börsengang des chinesischen Unternehmens Ant Group durch neue Regulierungsvorschriften zeigt, wie Regulierungsbehörden in China den zunehmenden Einfluss eines der größten chinesischen Technologiekonzerne durch Aktivitäten im Bereich der Finanzintermediation adressieren. In einer Pressemitteilung Ende 2020 nach einem Treffen mit den Verantwortlichen der Ant Group formulierten die chinesischen Regulierungsbehörden die Rückkehr zu seinen Wurzeln als Bezahldienst als eine von fünf Anforderungen an das Unternehmen. Darüber hinaus forderten die Behörden die Erfüllung der Regulierungsvorschriften im Bereich der Finanzdienstleistungen, eine angemessene Kapitalausstattung sowie Verbesserungen des Datenschutzes und der Corporate Governance (Chinesische Zentralbank 2020c). Die Erklärung der chinesischen Regulierungsbehörden verdeutlicht, dass die neuen Regulierungsvorschriften am umfangreichen Angebot von Finanzdienstleistungen des Unternehmens ansetzen.

Neben dem Zahlungsdienstleister Alipay gehören auch die folgenden Angebote zu den Tochterunternehmen der Ant Group. Zhima Credit bietet einen Service für unabhängige Bonitätsprüfungen auf Basis von Big-Data-Analysen der umfangreichen Kundeninformationen an. Darüber hinaus gehören zur Ant Group im Bereich der *Kreditvergabe* die MYbank, eine Online-Bank für Klein- und Kleinstunternehmen, sowie die beiden Plattformen Huabei für Konsumentenkredite mit Kreditkartencharakter und Jiebei für die Vergabe von Mikrokrediten. Im Bereich *Kapitalanlagen* bietet Yu’e Bao eine Cash-Management-Plattform, Versicherungsdienstleistungen auf der Plattform Xiang Hu Bao runden das Angebot ab (Ant Group 2021; Dathe und Helmold 2020). Dabei macht der Anteil des Umsatzes durch Kredite mittlerweile mehr als ein Drittel des Umsatzes der Ant Group aus und ist damit für das Unternehmen inzwischen ebenso wichtig wie der Umsatz aus dem Zahlungsdienst Alipay (Dathe und Helmold 2020; The Economist 2020c).

Konkret hatten die Regulierungsbehörden bereits im September 2020 einen Entwurf für neue Vorschriften veröffentlicht. Die Regeln, die am 1. November 2020 in Kraft traten, adressieren das zunehmende systemische Risiko durch die Aktivitäten großer Konzerne im Bereich der Finanzdienstleistungen. Unternehmen, die auf Basis eines Kriterienkatalogs als Finanzholding eingestuft werden, unterliegen demnach den gleichen Mindesteigenkapitalanforderungen wie traditionelle Banken (Chinesische Zentralbank 2020a; Wah et al. 2020). Zusätzlich schreiben ebenfalls ab dem 1. November 2020 gültige Regelungen zum Verbraucherschutz neue Anforderungen für Banken und Nicht-Bank-Zahlungsdienstleister^7^ vor (Chinesische Zentralbank 2020b). Diese Regelungen schließen an die zunehmend strengeren Vorschriften zur Regulierung des chinesischen Schattenbankensektors^8^ an, die chinesische Regulierungsbehörden seit 2017 umgesetzt haben (Internationaler Währungsfonds 2019b, S. 37–38; Sun 2019, S. 29).

Insbesondere der IWF betonte mehrfach, dass der Umgang mit Unternehmen wie der Ant Group für die Aufsichtsbehörden eine wachsende Herausforderung darstellt (Internationaler Währungsfonds 2017, S. 54–55, 2019c, S. 80–95). Unter anderem hatten chinesische Behörden 2018 Verbriefungen eingeschränkt, die durch das FSB als Teil des Schattenbankensektors im engen Sinne eingestuft werden (Financial Stability Board 2020c, S. 55–57). Diese Regulierungsänderung hatte ebenfalls Auswirkungen auf die Geschäftsaktivitäten der Ant Group, die ihre Kredite bis dahin in Form von Verbriefungen an Banken verkauft hatte (Financial Stability Board 2020c, S. 15; The Economist 2020a). Wie Abb. 1 für den Schattenbankensektor in China zeigt, hat sich das Verhältnis der Bilanzsumme des Sektors zum BIP dementsprechend seit 2017 stabilisiert und ist im Fall des Schattenbankensektors im engen Sinne sogar gesunken. Diese Entwicklung deutet an, dass die Regulierung erste Wirkungen zeigt. Speziell die fortschreitenden Änderungen der Geschäftsaktivitäten, die als Innovationen im Schattenbankensektor mit potenziellem Risiko für die Finanzstabilität eingestuft werden, erfordern darüber hinaus weitere Schritte, um die nachhaltige Stabilität des Finanzsystems sicherzustellen (Weltbank und Development Research Center of the State Council, Volksrepublik China 2019, S. 135; Financial Stability Board 2020c, S. 3–6).

Gerade weil die jüngsten Regulierungsvorschriften jedoch speziell die Ant Group ins Visier zu nehmen scheinen, ist alternativ eine politische Motivation als Erklärung für die rasche und konsequente Umsetzung der neuen Regulierungsvorschriften denkbar (Trivedi 2020; The Economist 2021). Jack Ma, der Gründer der Ant Group und ihr größter einzelner Gesellschafter mit einem Anteil von knapp neun Prozent, kritisierte im Oktober 2020 in einer Rede die Prinzipien der internationalen Bankenaufsicht. Vor dem Hintergrund von Unterschieden zwischen den Finanzsystemen weltweit, müsse die Aufsicht stärker zwischen unterschiedlichen Finanzmarktakteuren differenzieren (Dathe und Helmold 2020; Kang 2020; Trivedi 2020). Die Frage, ob die chinesischen Behörden aufgrund des Risikos der Ant Group für die Finanzstabilität oder aus politischen Gründen handelten, kann nicht eindeutig beantwortet werden. Während die politische Erklärung als chinaspezifisches Problem ausgeklammert werden kann, liefert der Fall in jedem Fall ein Beispiel für die Regulierung von BigTech mit potenziellen Implikationen für andere Wirtschafts- und Finanzsysteme weltweit (The Economist 2021).

## Kritische Würdigung des Regulierungsansatzes und Implikationen für die EU

Für die Beurteilung des Ansatzes der chinesischen Behörden zur Regulierung von Schattenbankenaktivitäten werden nun die folgenden Chancen und Herausforderungen erörtert: Gegen eine strenge Regulierung von BigTech sprechen die Vertiefung des Finanzmarktes durch technologiebasierte Finanzdienstleistungen, Vorteile dieser Unternehmen gegenüber traditionellen Banken durch ihre umfangreichen Kundeninformationen sowie ihre stabilisierende Wirkung auf die Volkswirtschaft durch geringe Ansteckungseffekte. Demgegenüber steht die Argumentation, dass BigTech durchaus ein systemisches Risiko für das Finanzsystem darstellen und durch Netzwerkeffekte in kurzer Zeit eine große Marktmacht erreichen. Regulierung kann in diesem Zusammenhang wesentlich zum Schutz der Konsumenten beitragen.

Indem BigTech Finanzdienstleistungen für Kreditnehmer zur Verfügung stellen, die bisher keinen Zugang zu Bankkrediten hatten, tragen sie zur *Vertiefung des Finanzmarktes* bei. Insbesondere in Ländern wie China, wo ein nennenswerter Anteil der Bevölkerung kein Bankkonto und keine Kreditkarte besitzt, lässt sich ein starkes Wachstum in der Kreditvergabe durch BigTech beobachten (Frost et al. 2019, S. 770–772; Hau et al. 2019, S. 62–64). Aber auch in entwickelten Ländern wie in Deutschland oder in den USA erfüllen technologiebasierte Finanzdienstleistungen eine durch traditionelle Banken nicht abgedeckte Nachfrage (De Roure et al. 2016, S. 16–17; Jagtiani und Lemieux 2018, S. 43; Tang 2019, S. 1900). Darüber hinaus ist ein technologiebasierter Algorithmus für die Kreditvergabeentscheidung in der Regel weniger anfällig für die Diskriminierung von Minderheiten nach Hautfarbe oder Herkunft des Kreditantragstellers (Bartlett et al. 2019, S. 21–22).

Auf Basis *umfangreicher Kundeninformationen* ermöglichen technologiebasierte Bonitätsprüfungen besonders zuverlässige Einschätzungen des Bonitätsrisikos. Dabei erhöht die fortgeschrittene Technologie nicht nur die Effizienz des Kreditvergabeprozesses, sondern ermöglicht auch eine günstigere Bereitstellung von Finanzdienstleistungen (Frost et al. 2019, S. 781–782; Financial Stability Board 2020a, S. 16–23). Indem ein Unternehmen, wie im Beispiel die Ant Group, auf die umfangreiche Datenbasis der E‑Commerce-Plattformen seines Mutterkonzerns zurückgreifen kann, erfolgt eine verlässlichere Einstufung der Kreditwürdigkeit potenzieller Kreditnehmer anhand von Big-Data-Analysen als dies bei traditionellen Banken möglich ist. Die geringere Ausfallquote der Kredite an mittelständische Unternehmen durch die Ant Group im Vergleich zu Banken könnte demnach auf den enormen Technologie- und Informationsvorteil der BigTech zurückzuführen sein (Frost et al. 2019, S. 781–782; The Economist 2021).

Eine potenzielle *stabilisierende Wirkung* der Finanzdienstleistungen von BigTech auf die Volkswirtschaft ist auf die geringe Korrelation der Kredite mit der nationalen Wirtschaftsleistung zurückzuführen (Gambacorta et al. 2020, S. 1). Anders als Bankkredite, die auf Änderungen von Immobilienpreisen reagieren und durch die Bedeutung von Immobilien als Sicherheiten den Zusammenhang zwischen der Realwirtschaft und dem Finanzsektor verstärken, sind die Ansteckungseffekte durch von Sicherheiten unabhängige Kredite geringer (Bernanke und Gertler 1989, S. 14–15; Gambacorta et al. 2020, S. 23). Zusätzlich können BigTech gerade in sich entwickelnden Märkten zum Aufbau einer fortgeschrittenen finanziellen Infrastruktur beitragen und so die Finanzstabilität nachhaltig verbessern (Financial Stability Board 2020a, S. 16–23).

Im Gegensatz dazu ist ebenso eine destabilisierende Wirkung von technologiebasierten Finanzdienstleistungen durch *systemisches Risiko* denkbar. Da BigTech Finanzdienstleistungen übernehmen, die andernfalls von Banken durchgeführt würden, sollten diese ebenso im Hinblick auf die finanziellen Risiken ihrer Aktivitäten untersucht werden. Dabei stellt die Regulierung als „Level Playing Field“ mit Anforderungen an alle Unternehmen, die Finanzdienstleistungen anbieten, eine besondere Herausforderung für die Regulierungsbehörden dar. Auf der einen Seite steht die Förderung eines innovativen und zukunftsfähigen Finanzsystems, auf der anderen Seite sollten alle Anbieter von Finanzdienstleistungen, die für identische Kunden mit ähnlichen Dienstleistungen ähnliche Risiken eingehen, möglichst einer ähnlichen Regulierung unterliegen (Carstens 2018, S. 4). Regulierung zur Verbesserung der Markteffizienz trägt dabei wesentlich zur Sicherung eines stabilen Finanzsystems bei (Diamond und Dybvig 1983). Andernfalls führt eine fehlende Kapitalunterlegung dazu, dass neue Marktteilnehmer im Finanzsystem potenziell erhöhte Risiken eingehen (Zetzsche et al. 2017, S. 35). Im Beispiel der Ant Group kommt hinzu, dass der Großteil der Kredite in den Bilanzen chinesischer Banken zu finden ist, und die Verflechtungen zwischen dem Schattenbankensektor und dem Bankensektor zusätzliche Risiken für die Finanzstabilität darstellen (Aldasoro et al. 2020, S. 68; The Economist 2020b).

Zusätzlich verfügen BigTech bereits über einen großen Kundenstamm, sodass durch Netzwerkeffekte eine große *Marktmacht* erreicht werden kann. Dieser Effekt wird auch dadurch verstärkt, dass die Attraktivität einer Plattform mit der Anzahl der Nutzer und dem Umfang der angebotenen Services steigt und sie dadurch eine immer größere Kundenbasis anzieht (Evans und Schmalensee 2010, S. 5–6). Sobald ein Unternehmen groß genug ist und ein Monopol aufgebaut hat, kann diese Position gegen Wettbewerber ausgenutzt werden, um weitere Gewinne zu erzielen (The Economist 2021). Im Fall der Ant Group ist bereits erkennbar, dass das Unternehmen aufgrund seiner Marktdominanz die Vertragskonditionen mit den kooperierenden Banken diktieren kann (Dathe und Helmold 2020).

Auf der Seite der Nutzer der Plattformen spielt zudem der *Schutz der Konsumenten* eine Rolle. Im Bereich der Finanzdienstleistungen stellt der Gläubigerschutz einen der zentralen Gründe für Regulierung dar. Kunden sind durch geringe finanzielle Bildung oder durch Unerfahrenheit möglicherweise nicht in der Lage, das Risiko von komplexen Finanzdienstleistungen adäquat zu beurteilen (Campbell 2016, S. 20). Eine angemessene Regulierung stellt die Integrität von Finanzdienstleistungen sicher, indem sie beispielsweise fordert, dass Finanzdienstleister ihre Kunden über potenzielle Risiken informieren. Zur Frage des Verbraucherschutzes allgemein kommt hinzu, dass gerade sensible Informationen eines besonderen Schutzes bedürfen. Sofern diese Informationen beispielsweise an Dritte weitergegeben werden, nimmt die Möglichkeit der Cyber-Kriminalität, der Daten-Manipulation und einer Überwachung von Individuen zu (The Economist 2020b).

Auf dieser Basis können die Auswirkungen einer Anwendung des Regulierungsansatzes der chinesischen Behörden auf die Finanzstabilität gegenübergestellt werden. Auf der einen Seite stellt die Anforderung, einen bestimmten Anteil der Kredite auf der eigenen Bilanz zu halten und Risiken mit Kapital zu unterlegen, sicher, dass ein Finanzdienstleister keine erhöhten Risiken eingeht („Skin in the game“). Damit trägt der Regulierungsansatz zu einer Reduzierung des systemischen Risikos bei (Wah et al. 2020). Erfahrungen aus der Finanzkrise 2008 haben gezeigt, dass andernfalls erhebliche Risiken für die Finanzstabilität entstehen können. Insbesondere bei großen und vernetzten Technologiekonzernen sollten Gefahren durch Ansteckungseffekte minimiert werden, da unerwartete Verluste negative Auswirkungen auf den Finanz- und Technologiesektor sowie die gesamte Wirtschaft haben können (Financial Stability Board 2017, S. 17–25; Zetzsche et al. 2017, S. 35). Auf der anderen Seite sollte eine potenzielle Schieflage dieser komplexen Konzerne berücksichtigt werden. Insbesondere da diese Konzerne so groß und vernetzt sind („Too big to fail“ und „Too interconnected to fail“), ergeben sich systemische Risiken und ein Konzern kann im Krisenfall auf staatliche Unterstützung hoffen. Neben Kapitalanforderungen und Aufsicht können Vorgaben für eine geordnete Abwicklung diese Herausforderungen adressieren (De la Mano und Padilla 2018, S. 511–515). Eine Regulierung auf Konzernebene könnte die Abwicklung im Krisenfall erschweren. Vor diesem Hintergrund stellt sich die Frage, ob nur Tochterunternehmen von Technologiekonzernen der Bankenregulierung und -aufsicht unterliegen sollten oder der gesamte Konzern.

Unter Berücksichtigung dieser Aspekte lassen sich Implikationen für die Regulierung des Schattenbankensektors in der EU ableiten. China zeigt, wie sich große Technologiekonzerne im Finanzbereich etablieren und zu einer Herausforderung für die Regulierung der Nicht-Bank-Finanzintermediation entwickeln könnten. Zunächst sind jedoch bislang keine mit der Ant Group vergleichbaren Unternehmen in dem Ausmaß in der EU aktiv. BigTech aus den USA bieten zwar Bezahldienste, eine umfassende Durchdringung des Marktes im Bereich der Kreditvergabe und der Kapitalanlage sind derzeit hingegen nicht absehbar.

Das Beispiel der Insolvenz der *Wirecard AG* zeigt jedoch, dass Probleme im Zusammenhang mit dem Angebot von Finanzdienstleistungen durch Technologiekonzerne die EU ebenfalls erreicht haben. Obwohl es sich in erster Linie um einen Betrugsfall handelt, lassen sich Lehren für die Regulierung und Aufsicht ziehen. So wird kritisiert, dass zwar Tochterunternehmen, wie in Deutschland die *Wirecard Bank AG*, der Aufsicht unterlagen, eine ganzheitliche Aufsicht über die Aktivitäten des Konzerns jedoch fehlte (Barba Navaretti et al. 2020, S. 8–12). Ähnlich wie die Ant Group betonte die Wirecard AG ihre Rolle als Technologieunternehmen, obwohl ihr Kerngeschäft im Bereich der Finanzdienstleistungen lag (Barba Navaretti et al. 2020, S. 11–12; Ant Group 2021).

Ein Blick auf die Automobilkonzerne in Deutschland verdeutlicht zudem die Notwendigkeit einer klaren Abgrenzung in der Definition einer Finanzholding, da diese Konzerne bei einer Anwendung des Regulierungsansatzes der chinesischen Behörden in der EU möglicherweise ähnlich behandelt werden müssten. Automobilkonzerne verfügen meist ebenfalls über ein Tochterunternehmen mit Vollbanklizenz und sind mit Aktivitäten im Bereich der Konsumentenkredite oder der Verbriefung von Leasingforderungen Teil des Schattenbankensektors (Deutsche Bundesbank 2014, S. 18). Anders als Technologiekonzerne, die Finanzdienstleistungen für eine deutlich größere Kundengruppe anbieten, sind die Finanzaktivitäten der Automobilkonzerne auf ein spezielles Segment im Finanzmarkt beschränkt. Negative Implikationen auf die Finanzstabilität werden daher als gering eingeschätzt (Financial Stability Board 2020c, S. 45).

Grundsätzlich ist eine Umsetzung vergleichbarer Regeln in der EU jedoch denkbar. Sofern das Angebot der Finanzdienstleistungen eines Technologieunternehmens mit dem einer Bank vergleichbar ist und eine umfangreiche Marktmacht gegeben oder bereits absehbar ist, erscheint eine Berücksichtigung in der Regulierung notwendig, um Finanzstabilität sicherzustellen (Zetzsche et al. 2017, S. 35; Dathe und Helmold 2020). Anders als bei kleinen innovativen, technologieorientierten Unternehmen, besitzen BigTech durch ihre originären Geschäftsaktivitäten bereits eine große Kundenbasis und können auf ihre Infrastruktur und ihren technologischen Vorteil gegenüber traditionellen Banken zurückgreifen, um in kurzer Zeit eine große Marktmacht aufzubauen. Durch fehlende Regulierung ihrer Geschäftsaktivitäten haben sie einen zusätzlichen Vorteil gegenüber Banken, der zunächst zum schnellen Wachstum des Sektors führt und auch im weiteren Verlauf den Wettbewerb verzerrt (Zetzsche et al. 2017, S. 35; Frost et al. 2019, S. 781–782). Insbesondere im Fall von ausgereiften Finanzdienstleistungen durch Technologieunternehmen, bei denen Risiken bereits abschätzbar sind, eignet sich eine Regulierung durch formelle Lizensierung oder rechtliche Vorschriften (Brummer und Yadav 2019, S. 282–297).

## Fazit und Ausblick

Die Diskussion der Implikationen des Regulierungsansatzes der chinesischen Behörden zeigt, dass die Frage, ob China an dieser Stelle ein Vorbild für die EU sein kann, maßgeblich von der Einschätzung des systemischen Risikos eines potenziell zu regulierenden Unternehmens abhängt. BigTech können zwar wesentlich zur Vertiefung und Stabilisierung des Finanzmarktes beitragen, es besteht jedoch die Gefahr, dass diese Unternehmen durch ihre Marktmacht ein erhebliches systemisches Risiko für das Finanzsystem darstellen. Gerade wenn ihre Tochterunternehmen vorwiegend in der Finanzbranche tätig sind, erscheint eine Regulierung auf Konzernebene unter Berücksichtigung der Größe und Vernetzung des Konzerns erforderlich, um Finanzstabilität sicherzustellen.

Da es in Europa bislang kein mit der Ant Group in China vergleichbares Unternehmen gibt und BigTech aus den USA hauptsächlich Bezahldienste anbieten, besteht kein akuter Handlungsbedarf. Dennoch sollten die europäischen Regulierungsbehörden die weitere Entwicklung genau beobachten und Maßnahmen für potenzielle Szenarien diskutieren. Ein solches Szenario könnte die Ausweitung des Angebots großer Technologiekonzerne im Bereich von Finanzdienstleistungen, wie etwa der Kreditvergabe oder der Kapitalanlage, sein. Ebenso wie die EU bereits US-amerikanische BigTech wie Facebook und Google auf Basis des geltenden Kartellrechts mit Bußgeldern belegte, erscheint eine Einschränkung der großen internationalen Technologiekonzerne nicht nur mit Blick auf faire Wettbewerbsbedingungen notwendig, um gegen die zunehmende Marktmacht dieser Konzerne vorzugehen (The Economist 2020d). Insbesondere wenn diese mit einem umfangreichen Angebot hauptsächlich in der Finanzbranche aktiv werden, sollte eine Berücksichtigung in der Regulierung mit Blick auf die Finanzstabilität in Betracht gezogen werden.

## Footnotes

1 Das Financial Stability Board (FSB) definiert Aktivitäten von Schattenbanken als Finanzintermediation durch Institutionen oder Aktivitäten außerhalb des traditionellen Bankensektors (Financial Stability Board 2012, S. 3). Seit 2018 verwendet das FSB den Begriff Nicht-Bank-Finanzintermediation, um seine zukunftsorientierte Betrachtungsweise des Sektors zu unterstreichen (Financial Stability Board 2019, S. 4).

2 Bhattacharya et al. (1998) geben einen Überblick über die Theorie der Bankenregulierung. Die wichtigsten Gründe für die Regulierung von Finanzintermediären sind die Stabilisierung des Systems beispielsweise durch die Vermeidung eines *Bank Runs* und potenziell daraus resultierende systemische Risiken (Diamond und Dybvig 1983; Saunders 1987), sowie die Notwendigkeit des Gläubigerschutzes, insbesondere vor dem Hintergrund der zunehmenden Komplexität der Finanzinstrumente (Campbell 2016, S. 28).

3 Die Definitionen eines entsprechenden Geschäftsmodells unterscheiden sich deutlich zwischen den in einer Umfrage des FSB befragten Ländern (Financial Stability Board 2019, S. 68–71). In Deutschland ist für Peer-to-Peer-Lending, definiert als die Vermittlung eines Kredits über eine Online-Plattform, für den Darlehensgeber eine Erlaubnis nach § 32 Absatz 1 KWG erforderlich (Deutsche Bundesbank 2020).

4 Die Gründe für die Finanzkrise sind vielfältig. Neben niedrigen Zinsen führten lockere Vergabestandards für Hypotheken in den USA zu zunehmenden Kreditvolumina mit zweifelhafter Bonität. In Form von Verbriefungen wurden diese Hypothekenkredite an sogenannte Zweckgesellschaften weitergegeben. Banken konnten somit nicht nur die Eigenkapitalunterlegung für diese Geschäfte umgehen, sondern trugen außerdem kaum Risiko (wenig „Skin in the game“). Da viele dieser komplexen Finanzprodukte durch Zweckgesellschaften oder andere Schattenbanken gehalten wurden, kann das Marktversagen im Herbst 2008 als *Run* auf den Schattenbankensektor charakterisiert werden (French et al. 2010, S. 3–32).

5 Der Schattenbankensektor gemäß der Definition des FSB umfasst das Volumen der Finanzaktiva der gesamten Nicht-Bank-Finanzintermediation (Financial Stability Board 2020c, S. 3).

6 Die Klassifikation der Schattenbankenaktivitäten im engen Sinne erfolgt anhand der Handlungsempfehlungen des FSB und beinhaltet unter anderem eine Kreditvergabe auf der Basis von Fristentransformation oder Hinweise auf eine Möglichkeit, bestimmte regulatorische Vorgaben für Banken zu umgehen („Regulatory Arbitrage“) (Financial Stability Board 2013b, S. 6–11, 2020c, S. 3–4).

7 In der Veröffentlichung der Maßnahmen für Verbraucherschutz von Finanzdienstleistungen der Chinesischen Zentralbank heißt es wörtlich: „Non-banking payment institutions (hereinafter referred to as payment institutions) that are legally established within the territory of the [People’s Republic of China] and provide payment services shall be subject to the provisions in the Measures“ (Chinesische Zentralbank 2020b, S. 2).

8 Sun (2019) liefert einen Überblick über die Besonderheiten des chinesischen Schattenbankensektors und diskutiert Herausforderungen für dessen Regulierung und Aufsicht.
